# Comparative analysis of human immune responses following SARS-CoV-2 vaccination with BNT162b2, mRNA-1273, or Ad26.COV2.S

**DOI:** 10.1038/s41541-022-00504-x

**Published:** 2022-07-06

**Authors:** Dominique J. Barbeau, Judith M. Martin, Emily Carney, Emily Dougherty, Joshua D. Doyle, Terence S. Dermody, Alejandro Hoberman, John V. Williams, Marian G. Michaels, John F. Alcorn, W. Paul Duprex, Anita K. McElroy

**Affiliations:** 1 Department of Pediatrics, University of Pittsburgh School of Medicine, Pittsburgh, PA USA; 2 Center for Vaccine Research, University of Pittsburgh School of Medicine, Pittsburgh, PA USA; 3 Institute of Infection, Inflammation, and Immunity, UPMC Children’s Hospital of Pittsburgh, Pittsburgh, PA USA; 4 Department of Microbiology and Molecular Genetics, University of Pittsburgh School of Medicine, Pittsburgh, PA USA

**Keywords:** Vaccines, Viral infection

## Abstract

SARS-CoV-2 vaccines BNT162b2, mRNA-1273, and Ad26.COV2.S received emergency use authorization by the U.S. Food and Drug Administration in 2020/2021. Individuals being vaccinated were invited to participate in a prospective longitudinal comparative study of immune responses elicited by the three vaccines. In this observational cohort study, immune responses were evaluated using a SARS-CoV-2 spike protein receptor-binding domain ELISA, SARS-CoV-2 virus neutralization assays and an IFN- γ ELISPOT assay at various times over six months following initial vaccination. mRNA-based vaccines elicited higher magnitude humoral responses than Ad26.COV2.S; mRNA-1273 elicited the most durable humoral response, and all humoral responses waned over time. Neutralizing antibodies against the Delta variant were of lower magnitude than the wild-type strain for all three vaccines. mRNA-1273 initially elicited the greatest magnitude of T cell response, but this declined by 6 months. Declining immunity over time supports the use of booster dosing, especially in the setting of emerging variants.

## Introduction

Two mRNA-based vaccines (BNT162b2 and mRNA-1273) and one adenovirus vector-based vaccine (Ad26.COV2.S) have been used in the US since EUA was granted for each (December 2020 for the mRNA vaccines and February 2021 for the adenovirus vaccine). While data have been published for each of these vaccines confirming safety, immunogenicity, and efficacy^1–9^, less data are available that compare vaccine-induced immune responses amongst the three vaccines using identical immunological assays^10–12^. In this study, we assessed a cohort of SARS-CoV-2 naive individuals who received BNT162b2, mRNA-1273, or Ad26.COV2.S vaccines for antigen-specific humoral and T cell immunity using identical immunologic assays to allow direct comparisons of the elicited responses. Emerging viral variants complicate the interpretation of vaccine efficacy and immunogenicity, and many studies to date have shown variable neutralizing ability in vaccinee serum for different viral variants^13–17^. Additionally, the role of T cells in providing protection from disease has been demonstrated in non-human primate studies^18^ and suggested in studies of humans^19^. To date, an immune correlate of protection has not been clearly established, however, neutralizing antibody titers have been suggested as a possible correlate, and indeed for mRNA-1273, at least two-thirds of vaccine efficacy has been attributed to neutralizing antibodies^19–21^. Waning of vaccine mediated immunity over time also became a pressing issue in the latter half of 2021 and led to the recommendation for additional vaccine doses.

## Results

Participant age, sex, and race were provided at the time of enrollment (Table 1). Information about co-morbidities was collected from the medical record or directly from study participants. All participants were healthy and without major co-morbidities unless otherwise noted. Blood was collected from all participants at four time points; at enrollment and approximately 1 month, 2 months and 6 months post initial vaccination (Table 2).

**Table 1:** Demographics of the study population.

Characteristic	No. (%)						
	BNT162b2 (*N* = 26)	*p* value^a^	mRNA-1273 (*N* = 24)	*p* value^a^	Ad26.COV2.S (*N* = 24)	*p* value^a^	Catchment area^b^ (*N* = 1,216,045)
Sex		0.0002		0.142		0.027	
Female	23 (88)		16 (67)		7 (29)		588,306 (51.7)
Male	3 (12)		8 (33)		17 (71)		627,739 (48.3)
Age group (yrs)^c^		0.296		0.710		0.689	
20–29	8 (31)		5 (21)		4 (17)		166,861 (17.4)
30–39	7 (27)		5 (21)		4 (17)		175,986 (18.4)
40–49	4 (15)		3 (13)		4 (17)		135,028 (14.1)
50–59	3 (12)		2 (8)		6 (25)		154,808 (16.1)
60–69	3 (12)		6 (25)		5 (21)		166,603 (17.4)
70–79	1 (4)		3 (13)		1 (4)		94,691 (9.9)
80+	0 (0)		0 (0)		0 (0)		64,625 (6.7)
Race/Ethnicity		0.414		0.003		0.0003	
White, non-Hispanic	24 (92)		19 (79)		17 (71)		948,157 (78.0)
Black, non-Hispanic	1 (4)		0 (0)		1 (4)		155,798 (12.8)
Hispanic	0 (0)		3 (13)		1 (4)		27,552 (2.3)
Asian/Pacific Islander, non-Hispanic	0 (0)		2 (8)		5 (21)		46,643 (3.8)
Multiple race/Other/Unknown	1 (4)		0 (0)		0 (0)		37,895 (3.1)

^a^One-way chi-squared goodness-of-fit tests comparing sample with catchment area demographics.

^b^Allegheny County, Pennsylvania. Source: United States Census Bureau, 2019 American Community Survey 1-year estimates.

^c^Age groups for catchment area based on estimates for population aged >19 y (*N* = 958,602).

**Table 2:** Intervals between vaccination and follow up visits for each vaccine group.

Median # Days (Interquartile Range)	BNT162b2	mRNA-1273	Ad26-COV2.S
Interval between vaccine 1 and visit 2	21 (17–21)	28 (27–29)	31 (28–35)
Interval between vaccine 1 and visit 3	45 (43–47)	63 (60–68)	60 (56–66)
Interval between vaccine 1 and visit 4	184 (182–189)	182 (179–191)	183 (179–189)
Interval between vaccine 2 and visit 3	24 (22–25)	36 (32–41)	
Interval between vaccine 2 and visit 4	162 (161–168)	155 (149–164)	

**Fig. 1:**
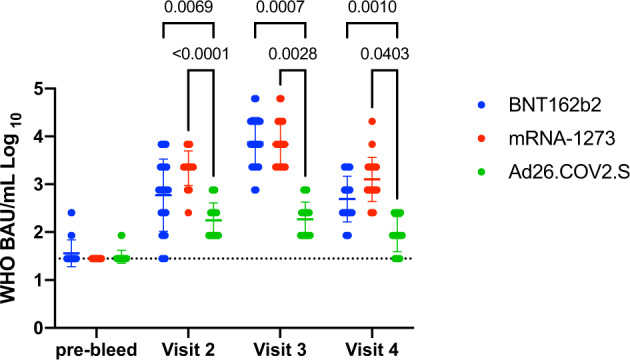
RBD ELISA titers amongst vaccine recipients. Plasma from each timepoint was tested by an RBD ELISA and then converted to WHO BAU/mL. Each data point is shown, the geometric mean and geometric standard deviation are plotted. The dotted line is the limit of detection of the assay (WHO BAU/mL of 28). Statistically significant differences using a mixed effects model with the Geisser-Greenhouse correction for unequal variance and Holm-Sidak multiple comparison test are noted by the respective p value.

All participants were tested by SARS-CoV-2 nucleoprotein (N) ELISA at enrollment, and 4 individuals were found to have N-specific ELISA titers greater than or equal to 900^22^. As this finding is consistent with prior SARS-CoV-2 infection, data from these participants were excluded from further analysis. One mRNA-1273 vaccine recipient relocated after enrollment precluding collection of additional data. After these exclusions, 26 BNT162b2 recipients, 24 mRNA-1273 recipients, and 24 Ad26.COV2.S recipients were included in the study. On the date of first vaccination (pre-bleed), 7 individuals had detectable, but low level, WT SARS-CoV-2 spike receptor binding domain (RBD) ELISA geometric mean titers (GMT) relative to a pre-pandemic normal human control group. ELISA titers were converted to WHO BAU/mL and there were no statistically significant differences in baseline WT RBD GMT between the vaccine groups (Fig. 1). At visit 2 (median 21–31 days post initial vaccination), most study participants had detectable antibody titers with the exception of two BNT162b2 recipients, one of whom was immunocompromised secondary to ongoing therapy for breast cancer with capecitabine. WT RBD GMT were significantly higher in BNT162b2 and mRNA-1273 recipients compared with Ad26.COV2.S recipients at all time points after initial vaccination. At visit 3 (median 45–63 days post initial vaccination), following a second dose for both of the mRNA-based vaccines, the BNT162b2 and mRNA-1273 GMT increased by approximately 1 Log_10_. RBD GMT observed in these cohorts correlated with those previously reported in immunogenicity studies of the various vaccines with Ad26.COV2.S achieving 2–3 Log_10_ GMT after one dose^6^, and mRNA-1273 and BNT162b2 achieving 3–5 Log_10_ GMT after completion of the two dose series^3,23^. Notably, there was a marked decline in titers at visit 4 (median of 182–184 days post initial vaccination) in all groups.

**Fig. 2:**
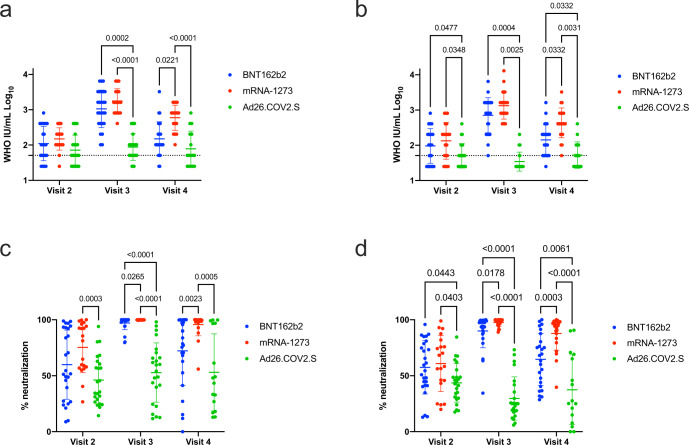
SARS-CoV-2 neutralization amongst vaccine recipients. Plasma from each timepoint was tested by a SARS-CoV-2 neutralization assay using a WT parental strain (**a**, **c**) or the Delta variant (**b**, **d**). FRNT_50_ data were converted to WHO IU/mL (**a**, **b**). Data are also shown as the percent of neutralization of input virus achieved at a 1:20 dilution of plasma (**c**, **d**). Each data point is shown, the geometric mean and geometric standard deviation (**a**, **b**) or mean and standard deviation (**c**, **d**) are plotted. The limit of detection for the FRNT_50_ assay is a WHO IU/mL titer of 51, indicated by a dotted line. Statistically significant differences using a mixed effects model with the Geisser-Greenhouse correction for unequal variance and Holm-Sidak multiple comparison test are noted by the respective *p* value.

Plasma samples from visits 2, 3 and 4 were tested in focus reduction neutralization 50% (FRNT_50_) assays using WT SARS-CoV-2 and the Delta variant. FRNT_50_ were converted to WHO IU/mL and were at or below the limit of detection (LOD = 51 WHO IU/mL) for a subset of participants after a single immunization and neutralization against WT virus did not differ significantly between vaccines (Fig. 2a, visit 2). However, both mRNA vaccines achieved higher neutralization GMT against the Delta variant than Ad26.COV2.S (Fig. 2b, visit 2). Following a second dose of either of the mRNA vaccines, neutralization GMT was significantly higher than those achieved with single dose Ad26.COV2.S vaccination for both WT SARS-CoV-2 (Fig. 2a, visit 3) and the Delta variant (Fig. 2b, visit 3). Following the booster dose, BNT162b2 and mRNA-1273 vaccination elicited similar neutralization GMT (Fig. 2a, b visit 3). At visit 4, recipients of mRNA-1273 had significantly higher WT (Fig. 2a) and Delta variant (Fig. 2b) neutralization GMT than recipients of either BNT162b2 or Ad26.COV2.S. For all samples, neutralization GMT were greater for WT SARS-CoV-2 than for the Delta variant (ranging from 1.2 to 2.4-fold higher) but did not achieve statistical significance.

Given the relatively low neutralization titers observed in some subjects, several observations did not meet the 50% focus-reduction threshold. Therefore, the data were are also depicted as the percent neutralization of input virus at the 1:20 dilution of plasma (Fig. 2c, d). This strategy allowed a more nuanced assessment of the capacity of individual plasma specimens to neutralize virus. E.g., two specimens at the 1:20 dilution may exhibit 5% and 40% neutralization; while both fail to achieve the FRNT_50_ threshold, there remains a clear difference in neutralization capability that is lost by reporting only FRNT_50_ titers. Following a single dose of vaccine, mRNA-1273 participants achieved higher percent neutralization at 1:20 dilution than those receiving Ad26.COV2.S (Fig. 2c, visit 2). Percent neutralization of WT and the Delta variant at 1:20 dilution elicited by either two dose mRNA vaccine series was higher in magnitude than that elicited by the single dose Ad26.COV2; mRNA-1273 also elicited higher percent neutralization than BNT162b2 (Fig. 2c, d, visit 3). This trend continued out to visit 4, with the exception that BNT162b2 GMT and percent neutralization waned sufficiently as to lose statistical significance compared to Ad26.COV2S for WT SARS-CoV-2 (Fig. 2a, c).

**Fig. 3:**
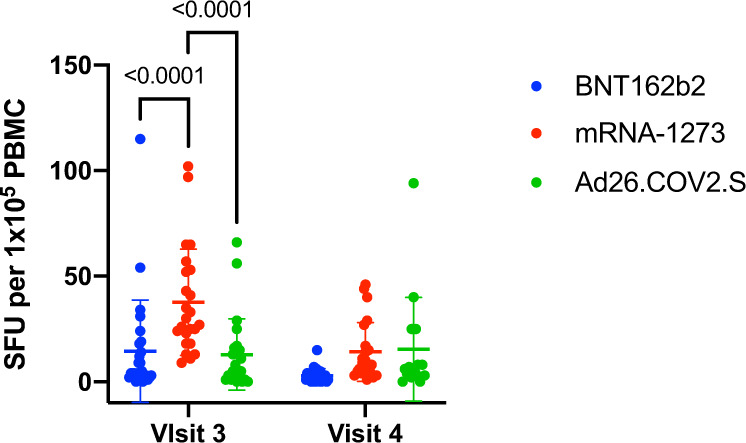
SARS-CoV-2 spike protein-specific T cell responses amongst vaccine recipients. PBMCs from the visit 3 and 4 timepoints were tested by an IFN-γ ELISPOT assay. Data are shown as spot forming units (SFU) per 100,000 PBMCs. Each data point is shown, the mean and standard deviation are plotted. Statistically significant differences using a mixed effects model with the Geisser-Greenhouse correction for unequal variance and Holm-Sidak multiple comparison test are noted by the respective *p* value.

WT SARS-CoV-2 spike glycoprotein-specific T cell responses were assessed using an IFN-γ ELISPOT assay with PMBCs from study participants at visits 3 and 4 (Fig. 3). mRNA-1273 recipients had a significantly higher magnitude of IFN-γ producing T cells (measured by spot forming units; SFU) following ex vivo stimulation of PBMCs with a total WT spike peptide mega pool when compared with BNT162b2 or Ad26.COV2.S recipients, but this effect waned over time and was not observed at visit 4, approximately 6 months after initial vaccination. No significant differences in T cell responses were apparent between the BNT162b2 and Ad26.COV2.S recipients at either time point. Notably, while BNT162b2 and mRNA-1273 recipient SFU/10^5^ PBMC declined from visit 3 to visit 4 (Wilcoxon rank sum test *p* = 0.0002 and *p* < 0.0001, respectively) those of Ad26.COV2.S were maintained (Wilcoxon rank sum test *p* = 0.8905).

## Discussion

### Comparative humoral immune responses in this study correlated with published efficacy data

Initial efficacy reports following phase III clinical trials against confirmed COVID-19 were 95.0%, 94.1%, and 66.9% for BNT162b2, mRNA-1273, Ad26.COV2.S, respectively^2,4,5^. Updated reports demonstrate decreasing efficacy at the 6-month timepoint against COVID-19 of 83.7%, 93.2% and 45.2% respectively^8,9,24^. These efficacy reports correlated with the humoral responses we observed over time in this study (Spearman r of 1, 0.9429, 0.8857 for WT RBD GMT, WT FRNT_50_ GMT and Delta FRNT_50_ GMT respectively). Notably, the reported efficacy did not correlate with cellular immune responses we observed over time (Spearman r of 0.2571 for SFU/10^5^ PMBC), consistent with reports that the majority of vaccine-mediated protection (at least in the case of mRNA-1273) can be explained by neutralizing antibody response^19^. Several studies have attempted to establish immunological correlates of protection following SARS-CoV-2 vaccination. In the COV002 trial (ChAdOx1 nCov019 vaccine), an RBD titer of 506 BAU/mL and a live virus neutralization titer of 247 IU/mL correlated with 80% protection from symptomatic infection with the Alpha variant^25^. In the COVE trial (mRNA-1273 vaccine), an RBD titer of 775 BAU/mL correlated with 90% protection from symptomatic infection with WT SARS-CoV-2^19^. Importantly, these assessments were made at 4 weeks post vaccination, so they represent the height of the immune response after vaccination. In a study of breakthrough infections following BNT162b vaccination in Italian health care workers, S1 Spike protein titers of 81–311 BAU/mL were noted in those who had breakthrough infections, and a marked decline in titers over time was noted^26^.

In this study, the mRNA-1273 vaccine achieved the greatest magnitude of spike-specific humoral immunity (FRNT_50_ GMT of 2422 IU/mL against WT SARS-CoV-2 at Visit 3) for the longest duration (FRNT_50_ GMT of 781 IU/mL against WT SARS-CoV-2 at Visit 4). This is congruous with a report of greater efficacy against infection and hospitalization for mRNA-1273 compared to BNTb162b, especially against the Delta variant^27^. It has also been reported that mRNA-1273 vaccination resulted in a greater magnitude of RBD binding capacity than BNTb162b^28^. The mRNA-1273 vaccine contains 100 μg of mRNA encoding the full-length, stabilized spike glycoprotein^2^, while the BNT162b2 vaccine contains 30 μg^4^. In our small cohort, BNT162b2 achieved a similar magnitude of antibody response after the second dose (FRNT_50_ GMT of 1931 IU/mL against WT SARS-CoV-2 at visit 3), but this waned more quickly (FRNT_50_ GMT of 325 IU/mL against WT SARS-CoV-2 at visit 4) than the mRNA-1273. In contrast, single dose Ad26.COV2.S had an FRNT_50_ GMT of 130 IU/mL against WT SARS-CoV-2 at visit 3, but this was maintained over time with an FRNT_50_ GMT of 157 IU/mL against WT SARS-CoV-2 at visit 4. The Ad26.COV2.S vaccine is administered at a dose of 5 × 10^10^ virus particles^5^. The adenovirus vector is replication-incompetent, thus, similar to the mRNA vaccines, only cells that take up the vaccine following inoculation will produce the SARS-CoV-2 spike glycoprotein. There is no amplification of these three vaccines in vivo, so not surprisingly, a dose-response effect was reported in early Phase I/II clinical trials of all three of the vaccine platforms^3,6,7,23^. However, as with all vaccines, the goal is to achieve a balance between reactogenicity and immunogenicity. Significant reactogenicity was reported in mRNA-1273 clinical trials and greater than 50% of study subjects reported a systemic adverse reaction of any grade after the first dose, and over 75% after the second dose^2^. The BNT162b2 vaccine also had significant reactogenicity^4^ but with lower frequency than mRNA-1273. Following single dose Ad26.COV2.S vaccination, over 60% of participants reported a systemic adverse reaction of any grade^5^. Significant reactogenicity in the setting of lower immunogenicity with the adenovirus vaccine might be attributable to pre-existing immunity to adenovirus or activation of innate immune pathways that differ from the mRNA vaccines.

### Antigen-specific T cells do not necessarily correlate with vaccine efficacy

The finding of significantly higher levels of WT spike-specific T cells following vaccination with mRNA-1273 than with either BNT162b2 or Ad26.COV2.S could be attributable to higher levels of antigen or to the dosing regimen. A notable feature of all three vaccines is the intracellular expression of viral antigen, which allows processing and presentation of spike glycoprotein-derived peptides in the context of MHC I and stimulation of virus-specific T cell responses. Intracellular antigen expression has historically been an advantage of viral-vectored vaccine platforms such as adenovirus-based vaccines. However, as our data demonstrate, mRNA vaccines can elicit equivalent (BNT162b2) or higher-magnitude (mRNA-1273) antigen-specific T cell responses compared to Ad26.COV2.S, but the mRNA vaccine induced T cell response was not durable over time, while the Ad26.COV2.S T cell response was maintained. Clinical trials indicated that BNT162b2 has an efficacy of 95% after the two-dose series, while Ad26.COV2.S efficacy was 66.9%. Since these two vaccines elicited similar T cell responses in IFN-γ ELISPOT assays, these data suggest that the T cell component of the immune response is probably not responsible for the enhanced protection afforded by BNT162b2 versus Ad26.COV2.S. However, the IFN-γ ELISPOT assay did not discriminate between CD4+ and CD8+ T cells, and thus, we cannot exclude the possibility that a specific T cell subset or function plays a role in protection, or that T cell mediated protection plays a role in the context of reduced humoral immunity.

### Could antigen-specific T cells provide a protective advantage against variants of concern?

One of the most pressing problems in the pandemic is the emergence of viral variants of concern (VOC). Notably, the efficacy data reported out to 6 months following vaccination includes data collected through March of 2021 for BNT162b2 and mRNA-1273 and July of 2021 for Ad26.COV2.S, hence these efficacy data do not reflect infection with the Delta or Omicron variants. Serum from vaccine recipients appears to have some neutralizing activity against the variants reported thus far, despite a reduction in viral neutralizing capacity in some cases^14,17,29–32^. Additionally, vaccinated individuals appear to be largely protected from severe disease and hospitalization following infection with VOC, although the evolving data on the Omicron variant demonstrates reduced vaccine efficacy, even in boosted individuals^33–37^. Studies of influenza vaccination suggest that heterologous protection is provided by virus-specific T cell immunity^38^. Most SARS-CoV-2 T cell epitopes are largely conserved between the variants, and there is preservation of cross-reactive cellular immunity over time^36,39,40^; even unexposed individuals have some virus-specific T cells due to cross-reactivity with epitopes conserved among common human coronaviruses^41^. Given the existence of virus-specific, cross-reacting T cells, we and others have hypothesized that increased protection from disease caused by variants could be provided by vaccines that stimulate strong T cell responses in addition to strong humoral responses. In that case, our data indicate that mRNA-1273 could potentially provide better protection from variants than either BNT162b2 or Ad26.COV2.S. Further research will be required to test this hypothesis.

Limitations of our study include that it was an observational cohort study without randomization, and participants receiving the various vaccines were not matched for demographics. As a result, there were sex and age differences and different racial distributions between the groups. The different dosing regimens as approved by the FDA under EUA resulted in slightly different intervals post vaccination for sampling and could have influenced the observed immunogenicity of the various vaccines. Additionally, our study lacks data on heterologous regimens, or two dose Ad26.COV2.S regimens. Despite these limitations, our study provides a direct side-by-side assessment of the elicited immune responses for 3 different vaccines, when comparative assessments between vaccines is limited in the current literature^12^. Our humoral immune measures correlated with published efficacy data for these three vaccines and demonstrated waning of effectiveness over time. These data support the conclusion that vaccine boosting is necessary to sustain high levels of antigen specific immunity, and to potentially provide protection against emerging VOCs.

## Methods

### Human subjects research

A convenience sample of participants 18 years of age and older were prospectively enrolled if they were planning to receive two doses of mRNA-1273 or BNT162b2, or a single dose of Ad26.COV2.S under FDA EUA between December 2020 and March 2021 either in an occupational or a community setting. After obtaining written informed consent, whole blood was obtained in cell preparation tubes (BD) on the day of enrollment/first vaccination, and during follow up visits. Plasma and peripheral blood mononuclear cells (PBMCs) were isolated and cryopreserved using standard methods. Institutional Review Board approval was provided by the University of Pittsburgh Human Research Protection Office and informed consent was obtained from all participants.

### Enzyme-linked immunosorbent assay (ELISA) and focus reduction neutralization assays (FRNT)

ELISA assays were conducted as follows. Briefly, MaxiSorp^TM^ 96-well plates (Thermofisher) were coated with SARS-CoV-2 RBD-His or SARS-CoV-2 N-His protein at 50 ng per well diluted in PBS and incubated at 4 °C overnight. Following removal of coating solution, plates were blocked with blocking buffer (5% non-fat milk in PBS with 0.1% Tween-20, PBST) and incubated at 37 °C for 1 h. Three-fold serial dilutions of samples were prepared in blocking buffer, then incubated on plates at 37 °C for 2 h. Plates were washed three times with PBST followed by incubation with donkey anti-human IgG horseradish peroxidase (HRP) conjugated secondary antibody (Jackson ImmunoResearch, Cat # 709–035–149) diluted 1:10,000 in blocking buffer at 37 °C for 1 h. Plates were washed again prior to the addition of TMB peroxidase substrate mix (Seracare) and incubated at room temperature (RT) for 5 min. TMB stop solution (Seracare) was added and the optical density (OD) at 450 nm was measured using a Molecular Devices SpectraMax 340PC Microplate Reader. For FRNT assays, two-fold dilution series of heat-inactivated plasma samples in DMEM-10 starting at 1:5 were incubated with an equal volume of 2 × 10^3^ FFU/mL of parental SARS-CoV-2 or Delta variant for 1 h at 37 °C. Parental SARS-CoV-2 (WT) was a clinical isolate obtained in summer of 2020 and has only a D614G mutation in the spike protein compared to Wuhan-Hu-1 strain (GenBank MN908947.3). The Delta variant (NR-55611) was obtained from BEI and grown in Vero cells expressing TMPRSS2 and hACE2. Spike protein sequence was confirmed prior to use.100 µL of the plasma/virus mixture was inoculated onto Vero E6 (WT SARS-CoV-2) or Vero cells expressing TMPRSS2 and hACE2 (Delta variant) and allowed to adsorb for 1 h at 37 °C before being replaced with a 1.5% carboxymethylcellulose overlay. Approximately 18 h later, plates were fixed in 10% formalin and then permeabilized with 0.1% Triton X-100 in phosphate buffered saline (PBS) for 10 min at RT. Plates were washed in PBST (PBS with 0.1% Tween-20) and then blocked with 5%-milk PBST for 1 h at RT. Primary antibody—a rabbit-anti-SARS-CoV-2 N (Genscript, custom)—was applied at 1:3000 diluted in block for 1 h at RT. Plates were washed twice with PBST before a 1-h RT incubation with peroxidase-conjugated goat-anti-rabbit IgG (Jackson ImmunoResearch, Cat # 111–035–144) diluted 1:1000 in block. The PBST wash was repeated, and TMB-H (MossBio) was used to develop foci. Foci were imaged and counted using a CTL ImmunoSpot reader. Samples with ELISA or FRNT_50_ titers below the limit of detection (LOD = 100 or 10 respectively) were assigned a value of 99 (28 WHO BAU/mL) or 5 (25 WHO IU/mL) respectively for graphical depiction and statistical analysis.

### ELISPOT assays

PMBCs were incubated for 24 h with 2 μg/mL of a SARS-CoV-2 complete spike glycoprotein mega pool consisting of 15-mer peptides overlapping by 11 residues based upon the original Wuhan-Hu-1 strain (GenBank MN908947.3) (Miltenyi) with 1 × Cell Activation Cocktail (without Brefeldin A, BioLegend) or media alone. Human IFN-γ ELISPOT assays were conducted according to the manufacturer’s instructions (Mabtech).

### Data analysis

Graphpad Prism was used for statistical analysis and to prepare figures. Four samples were missing from visit 2 and 2 from visit 4 for mRNA-1273 recipients; 1 sample was missing from visit 4 for BNT162b recipients; 9 samples were missing from visit 4 for Ad26.COV2.S recipients due to participant dropout. Samples were available for all other participants at all time points. To analyze ELISA, neutralization and ELISPOT data, a mixed-effects model with the Geisser-Greenhouse correction and Holm-Sidak’s multiple comparison test was used. Sex, age, and racial/ethnic distributions of participants were compared with those of the catchment area using one-way chi-squared goodness-of-fit tests. RBD ELISA and FRNT_50_ data were converted to the WHO International standard (IS) using the Human SARS-CoV2 Serology Standard Lot COVID-NS01097 (Frederick National Laboratory) according to the following formula: Sample titer/Standard titer × 764 BAU/mL (for ELISA) or 813 IU/mL (for FRNT).

### Reporting summary

Further information on research design is available in the Nature Research Reporting Summary linked to this article.

## Supplementary information

REPORTING SUMMARY

## Data Availability

All data are included in the manuscript. Any requests for additional details are welcome and can be directed towards the corresponding authors.
