# Supplementary material for: Comparative analysis of human immune responses following SARS-CoV-2 vaccination with BNT162b2, mRNA-1273, or Ad26.COV2.S
Source: NPJ Vaccines. 2022 Jul 6;7:77. doi: 10.1038/s41541-022-00504-x (PMC9258461; doi:10.1038/s41541-022-00504-x)
Supplement: Supplementary file 1 — REPORTING SUMMARY [file 41541_2022_504_MOESM1_ESM.pdf]

nature portfolio

Corresponding author(s): Anita McElroy
Judith Martin

Last updated by author(s): Apr 26, 2022

Reporting Summary

Nature Portfolio wishes to improve the reproducibility of the work that we publish. This form provides structure for consistency and transparency in reporting. For further information on Nature Portfolio policies, see our Editorial Policies and the Editorial Policy Checklist.

Statistics

For all statistical analyses, confirm that the following items are present in the figure legend, table legend, main text, or Methods section.

n/a	Confirmed	
☐	☒	The exact sample size (n) for each experimental group/condition, given as a discrete number and unit of measurement
☐	☒	A statement on whether measurements were taken from distinct samples or whether the same sample was measured repeatedly
☐	☒	The statistical test(s) used AND whether they are one- or two-sided<br>*Only common tests should be described solely by name; describe more complex techniques in the Methods section.*
☒	☐	A description of all covariates tested
☐	☒	A description of any assumptions or corrections, such as tests of normality and adjustment for multiple comparisons
☐	☒	A full description of the statistical parameters including central tendency (e.g. means) or other basic estimates (e.g. regression coefficient) AND variation (e.g. standard deviation) or associated estimates of uncertainty (e.g. confidence intervals)
☒	☐	For null hypothesis testing, the test statistic (e.g. F, t, r) with confidence intervals, effect sizes, degrees of freedom and P value noted<br>*Give P values as exact values whenever suitable.*
☒	☐	For Bayesian analysis, information on the choice of priors and Markov chain Monte Carlo settings
☒	☐	For hierarchical and complex designs, identification of the appropriate level for tests and full reporting of outcomes
☒	☐	Estimates of effect sizes (e.g. Cohen's d, Pearson's r), indicating how they were calculated

Our web collection on statistics for biologists contains articles on many of the points above.

Software and code

Policy information about availability of computer code

Data collection	MS Excel
Data analysis	GraphPad Prism or MS Excel

For manuscripts utilizing custom algorithms or software that are central to the research but not yet described in published literature, software must be made available to editors and reviewers. We strongly encourage code deposition in a community repository (e.g. GitHub). See the Nature Portfolio guidelines for submitting code & software for further information.

Data

Policy information about availability of data

All manuscripts must include a data availability statement. This statement should provide the following information, where applicable:

- Accession codes, unique identifiers, or web links for publicly available datasets
- A description of any restrictions on data availability
- For clinical datasets or third party data, please ensure that the statement adheres to our policy

All data are included in the manuscript. Any requests for additional details are welcome and can be directed towards the corresponding authors.

Field-specific reporting

Please select the one below that is the best fit for your research. If you are not sure, read the appropriate sections before making your selection.

☒ Life sciences ☐ Behavioural & social sciences ☐ Ecological, evolutionary & environmental sciences

For a reference copy of the document with all sections, see nature.com/documents/nr-reporting-summary-flat.pdf

Life sciences study design

All studies must disclose on these points even when the disclosure is negative.

Sample size	It was estimated that a sample size of 20 per group would give 80% power to detect a difference in RBD titer of 2 dilutions between groups with an alpha of 0.05.
Data exclusions	Participants with detectable baseline N ELISA titers were excluded on the basis that they were not naive.
Replication	All assays were performed in duplicate.
Randomization	There was no randomization in this observational cohort study.
Blinding	Neither investigators nor participants were blinded.

Reporting for specific materials, systems and methods

We require information from authors about some types of materials, experimental systems and methods used in many studies. Here, indicate whether each material, system or method listed is relevant to your study. If you are not sure if a list item applies to your research, read the appropriate section before selecting a response.

Materials & experimental systems

n/a	Involved in the study	
☐	☒	Antibodies
☐	☒	Eukaryotic cell lines
☒	☐	Palaeontology and archaeology
☒	☐	Animals and other organisms
☐	☒	Human research participants
☒	☐	Clinical data
☒	☐	Dual use research of concern

Methods

n/a	Involved in the study	
☒	☐	ChIP-seq
☒	☐	Flow cytometry
☒	☐	MRI-based neuroimaging

Antibodies

Antibodies used	Custom rabbit anti-SARS-CoV-2 N generated commercially by Genscript.
Validation	Specificity confirmed by testing on uninfected and SARS-CoV-2 infected Vero E6 cells

Eukaryotic cell lines

Policy information about cell lines

Cell line source(s)	ATCC
Authentication	Not authenticated.
Mycoplasma contamination	Cell lines tested negative for mycoplasma contamination.
Commonly misidentified lines (See ICLAC register)	n/a

Human research participants

Policy information about studies involving human research participants

Population characteristics	All participants were adults of both sexes and varying races.

Recruitment	Individuals who were receiving any of the vaccines either in the community or the workplace were invited to participate.
Ethics oversight	University of Pittsburgh approved the study.

Note that full information on the approval of the study protocol must also be provided in the manuscript.
